# Epiphytic microflora and mycotoxin content in meadows–Is plant biodiversity affecting fungal contamination?

**DOI:** 10.1371/journal.pone.0288397

**Published:** 2023-09-14

**Authors:** Ivana Kolackova, Barbora Smolkova, Jiri Skladanka, Petr Kouril, Eva Hrudova

**Affiliations:** 1 Department of Animal Nutrition and Forage Production, Faculty of AgriSciences, Mendel University in Brno, Brno, Czech Republic; 2 Department of Agrochemistry, Soil Science, Microbiology and Plant Nutrition, Faculty of AgriSciences, Mendel University in Brno, Brno, Czech Republic; 3 Department of Crop Science, Breeding and Plant Medicine, Faculty of AgriSciences, Mendel University in Brno, Brno, Czech Republic; Universitat Jaume 1, SPAIN

## Abstract

Ecosystem services are an important aspect of grasslands utilization; however, they are often contradictory to their main purpose, which is a production of good quality and safe feed. In this study, we evaluated the difference between grass monocultures and species-rich mixtures in terms of epiphytic microflora and mycotoxin contamination levels. We hypothesized that higher species diversity would lead to higher microbial counts, which could lead to higher mycotoxin contamination risk. Differences in epiphytic fungal, yeast and total amount of microorganisms (CFU g ^-1^) depending on the species diversity in the field has been evaluated by cultivation method. Concentration of deoxynivalenol (DON), zearalenone (ZEN) and aflatoxin B1 (AFB1) was measured by ELISA. Results are suggesting that higher total amount of microorganisms were found in monocultures, however, fungal and yeast counts were higher in species-rich mixtures. Higher species diversity of grasses was related to higher total microbial count (TMC) and yeast colonization of phyllosphere. Our results suggest higher risk of fungal phyllosphere colonization of species-rich mixtures with higher biodiversity and therefore higher risk of mycotoxin contamination of such feed.

## Introduction

Grasslands can be found all over the world and are one of the most abundant types of ecosystems on the planet. With the total area of 49×10^6^ km^2^, they serve a crucial role in feeding world’s animal production [1]. However, production of potentially high-quality and low-cost fodder is only one of many important functions. Non-productive aspects of grasslands such as anti-erosion, phytosanitary and structure-forming functions are getting to the forefront of public perception in the recent years as well as the issue of maintaining biodiversity in agricultural land [2, 3]. These aspects are, however, only secondary for most farmers, and therefore the rise in practical public policies and potential subsidies is unavoidable for changing farmers’ mindset to focus not only on yield of forage production, but on ecosystem services as well [2]. The biggest issue with the wider use of flowering meadows for feeding delves in the insufficient information about quality of such feed.

Quality of the produced feed depends mainly on the quality of inputs, e.g. forage grasses and legumes. Fodder quality has a decreasing tendency each month throughout the year with lowest quality in the oldest fodder stands. Study of Kononenko and Burkin [4] shows, that the highest values of secondary metabolites, which are a common cause of health complications and toxicosis in cattle, occur more frequently in the autumn. Additionally, it is possible to see the emergence of mycotoxins in the areas where they were not detected in the past as a result of climate change [5–7].

Levels of mycotoxin contamination of forage stands changes depending on the dominant species in the mixture. Study of Kononenko and Burkin [8] states, that mycotoxins were present in all mixtures. Furthermore, they were detected in at least 50% of the samples. In the mixtures where legume *Vicia* sp. was dominant was deoxynivalenol (DON) absent. Another study adds that agroforestry can change the abundance of soil bacteria and fungi in comparison with a monoculture and open grassland [9]. This suggests that number of grown species may affect epiphytic microbiota as well.

Mycotoxicosis is often difficult to diagnose due to its non-specific symptoms and symptoms’ similarity with other clinical diagnoses. Therefore, the mycotoxins’ effects on animal health can often escalate to death of an animal due to a slow diagnosis and often incorrect treatment. This goes hand in hand with the welfare aspect of animal husbandry. Subsequently it is important to note the economic impact on production by organ damage and decrease in weight gain, feed efficiency and reproduction rate [10]. Clinical symptoms such as anorexia, impaired rumen functionality, ankle adduction, posterior paralysis, dairy cow syndrome etc. often occur [11].

Research interest in mycotoxin presence in fresh forage is limited in comparison with mycotoxin contamination of cereals [12]. In the light of current information, it is important to evaluate the quality of species-rich mixtures in comparison to monocultures for increasing the their demand and growing area. We postulated that in diverse ecosystem, such as species-rich mixtures, will be microorganisms, and especially fungi, more abundant. This, moreover, may lead to higher levels of mycotoxin contamination in comparison with monocultures.

## Material and methods

**Fig 1:**
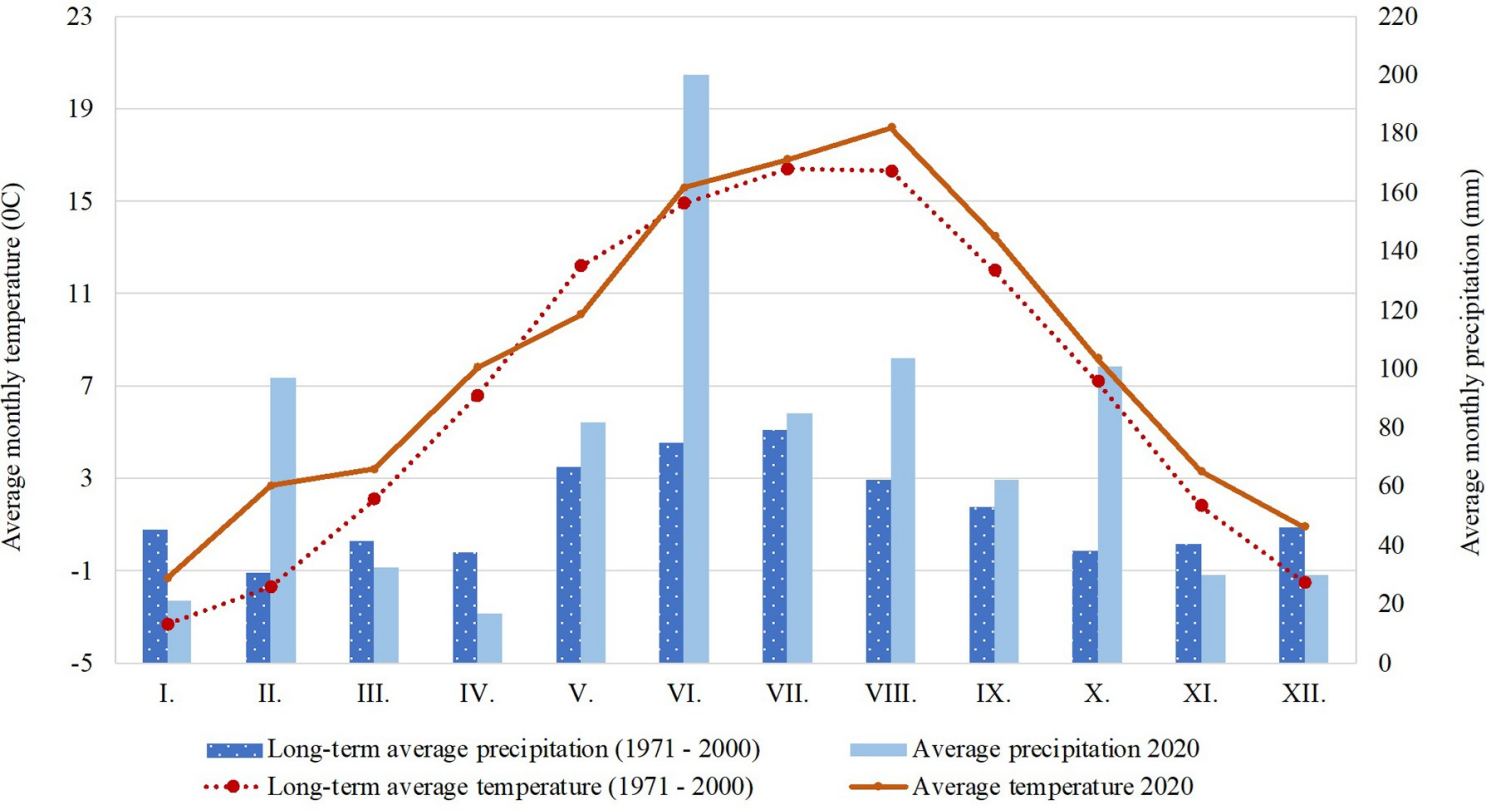
Average monthly temperature and precipitation in the experimental location.

The experiment was established in 2018 on Mendel University in Brno’s Research station Vatín, located in in the Bohemian-Moravian Highlands (49°52’ N, 15°96’ E). Site is located 560 m above sea level. Average yearly precipitation is 617 mm and average yearly temperature is 6.9°C. Monthly weather conditions can be found in Fig 1. Soil class on the site is loamy-sand, soil type is cambisol on the diluvium of piotic orthogenesis. Small plots used in the experiment had 10 m^2^ (1,25x8 m), no fertilization was applied in any of the variants. Each variant was established in triplets in Latin square design. Sampling was done 8. 10. 2020.

Following types of 2-year old stands were studied:

- Grass monocultures: *Festuca arundinacea* Schreb. (in the varieties ’Prosteva’, ’Kora’, ’Aprilia’, ’Philia’) and *Lolium perenne* L. (in the variety ’Promed’)
- Species-rich meadow mixtures comprising up to 27 plant species (Table 1):
  ⚬ Mixture 1: 0% grasses + 40% legumes + 60% forbs
  ⚬ Mixture 2: 70% grasses + 10% legumes + 20% forbs
  ⚬ Mixture 3: 90% grasses + 3% legumes + 7% forbs

**Table 1:** Species-rich mixtures’ seed components.

Botanical species	Variety	Percentage of component in the mixture (%)		
		Mixture 1	Mixture 2	Mixture 3
*Agrostis capillaris* L.			2.3	3.0
*Anthoxanthum odoratum* L.			3.1	4.0
*Anthyllis vulneraria* L.	Pamir	5.3	1.3	0.4
*Arrhenantherum elatius* (L.) J. Presl et C. Presl			2.3	3
*Bromus erectus* Huds.			7.8	10
*Carum carvi* L.	Prochan	1,7	0.6	0.2
*Centaurea jacea* L.		1.7	0.6	0.2
*Cynosurus cristatus* L.	Rožnovská		6.2	5
*Fagopyrum esculentum* Moench	Zita	17.1	5.7	2
*Festuca pratensis* Huds.	Otava		6.2	8
*Festuca rubra commutata*	Zulu		5.4	5
*Festuca rubra rubra*	Tagera		11.7	15
*Festuca rubra trichophylla*	Viktorka		3.9	7
*Festuca trachyphylla* (Hack.) R. P. Murray	Dorotka		7.8	18
*Knautia arvensis* (L.) Coulter		4.3	1.4	0.5
*Leucanthemum vulgare* Lam.		8.6	2.8	1
*Lolium perenne* L.	Jozífek		1.6	2.0
*Lotus corniculatus* L.	Leo	26,7	6.7	2.0
*Onobrychis viciifolia* Scop.	Višňovský	6.7	1.7	0.5
*Phacelia tanacefolia* Benth.	Větrovská	17.1	5.7	2
*Phleum pratense* L.	Sobol		1.6	2
*Poa pratensis* L.	Balin		7.8	10
*Salvia pratensis* L.		6	2	0.7
*Sanguisorba minor* Scop.		2.6	0.9	0.3
*Silene vulgaris* (Moench) Garcke		0.9	0.3	0.1
*Trifolium pratense* L.	Start	1.3	0.3	0.1
*Trisetum flavescens* (L.) P. Beauv.	Horal		2.3	3

Botanical composition of the mixture was species-wise the same as the botanical composition of experimental plots after two years after the establishment (during the sampling). Weed species that were found on experimental variants can be found in Table 2. The overall weed species’ percentage was <1% in monocultures and Mixture 1. The weed species’ percentage was <5% in Mixtures 2 and 3. Therefore their effect on the experiment can be considered negligible.

**Table 2:** Weed occurrence in experimental variants.

	'Prosteva'	'Kora'	'Aprilia'	'Philia'	'Promed'	Mixture 1	Mixture 2	Mixture 3
***Taraxacum* sect. *Ruderalia* Kirschner, H. Ollgaard & Štěpánek**	+	+	+	+	-	+	+	+
***Cerastium arvense* L.**	-	-	-	-	+	+	+	+
***Lamium album* L.**	+	+	+	+	+	-	-	-
***Poa annua* L.**	-	-	-	-	+	+	+	+
***Veronica arvensis* L.**	+	+	+	+	+	+	+	+
***Veronica chamaedrys* L.**	+	-	-	-	-	-	-	-
***Spergula arvensis* L.**	-	-	-	-	-	+	+	+
***Galeopsis tetrahit* L.**	-	-	-	-	-	+	+	+
***Stellaria media* (L.) Vill.**	-	-	-	-	-	+	+	+
***Echinochloa crus-galli* (L.) P. Beauv.**	+	+	+	+	+	+	+	+

Symbol “+” marks occurrence of the weed species in the variant. No occurrence is marked with “-“.

All analyses were done in specialised laboratory at Mendel University in Brno (49°21’ N, 16°61’ E). For microbial analysis 10 g of fresh biomass were used. The sample was homogenized in sterile polyethylene bags on STOMACHER (Interscience, France) with 90 ml of sterile saline for 1 minute. From the inoculum a series of 10-fold dilutions were prepared. Following groups of microorganisms were cultivated: i) total microbial count (TMC) on Plate Count Agar at 30°C for 72 h; ii) fungi (yeasts and other fungi) on Chloramphenicol Glucose Agar at 25°C for 120 h. Cultivation media were prepared according to the producers’ instructions (BiokarDiagnostics, Allone, France). ColonyStarcolony counter (Funke Gerber, Berlin, Germany)withpressure-sensitive automatic counter and illuminated counting platewas used for countingof CFUs(colony forming units).The result was expressed asa number ofCFUsper gram offreshsample.

Biomass samples for mycotoxin analysis were prepared by drying fresh biomass at 60°C. Pulverisette laboratory cutting mill (Fritsch, Idar-Oberstein, Germany) for preparation of samples (<1 mm particles) was used. ELISA was used for the quantitative analysis of mycotoxins. Samples were prepared according to the test kits’ instructions (MyBioSource, San Diego, CA, USA). Kits’ detection limits were 0.03 μg kg^-1^ for deoxynivalenol (DON), 0.18 μg kg^-1^ for zearalenone (ZEN) and 0.03 μg kg^-1^ for aflatoxin B1 (AFB1). Wavelenght in Synergy HTX Multi-Mode Microplate Reader (BioTek, Winooski, VT, USA) was adjusted to 450 nm. Concentration of DON, ZEN and AFB1 was measured.

Data were evaluated by StatSoft Statistica 12.0 (TIBCO Software Inc., Palo Alto, CA, USA). Shapiro-Wilk test for normality was used. Normal data was evaluated by single-factor ANOVA and post-hoc Scheffé test, otherwise evaluation was done by Kruskal-Wallis ANOVA or Mann-Whitney test. Significant differences were accepted if *p* < 0.05.

## Results

There were no differences observed between TMC found in monocultures and species-rich mixtures, average values measured were3.02x10^9^ CFU g ^-1^ and1.72x10^9^ CFU g ^-1^ (Table 3).

**Table 3:** Average microbial colonization and mycotoxin contamination depending on diversity of botanical species in the sample.

	Mixtures (n = 9)		Monocultures (n = 15)	
	Mean value	SD	Mean value	SD
Total amount of microorganisms (log CFU g ^-1^)	9.24 ^a^	9.00	9.48 ^a^	9.74
*Saccharomycotina* (log CFU g ^-1^)	6.73^a^	6.65	4.68 ^b^	4.59
Other fungi (CFU g ^-1^)	6.68 ^a^	6.69	5.39 ^b^	5.44
Deoxynivalenol (μg kg^-1^)	1.76 ^a^	1.31	<LOQ	<LOQ
Aflatoxin B1(μg kg^-1^)	0.03^a^	0.03	0.05 ^b^	0.01
Zearalenone (μg kg^-1^)	0.87^a^	0.34	0.42 ^b^	0.20

Results are expressed as a mean and standard deviation (SD). Mycotoxin concentration marked as <LOQ were under the limit of quantification (DON < 0.03 μg kg^-1^). Mean values are statistically significant at p < 0.05, when values in columns are marked with a different letter in the upper index.

Significantly higher amounts of both *Saccharomycotina* (yeasts) and other fungi (OF)were found in mixtures (yeasts 5.39x10^6^ CFU g ^-1^ and OF 4.82x10^6^ CFU g ^-1^) in comparison to monocultures (yeasts 4.81x10^4^CFU g ^-1^ and OF 2.44x10^5^CFU g ^-1^).

The highest TMC present on the above-ground biomass was observed in perennial ryegrass (1.37x10^10^ CFU g ^-1^). Furthermore, species-rich mixtures and ryegrass were more colonized by microorganisms than monocultures of tall fescue (Table 4). Significantly higher values were measured in Mixture II and III, there were no differences between Mixture I and fescues. The lowest microbial colonization was observed in ’Aprilia’ (4.09x10^7^ CFU g ^-1^).

**Table 4:** Average microbial colonization depending on botanical species.

		Total amount of microorganisms (log CFU g ^-1^)	*Saccharomycotina* (log CFU g ^-1^)	Other fungi (log CFU g ^-1^)
*Festuca arundinacea* ’Prostela’ (n = 3)	**Mean**	8.13 ^a^	4.35 ^a^	4.92 ^a^
	**SD**	6.66	2.83	4.22
*Festuca arundinacea* ’Aprilia’ (n = 3)	**Mean**	7.61 ^a^	4.53 ^a^	7.61 ^a^
	**SD**	7.13	3.50	3.06
*Festuca arundinacea* ’Philia’ (n = 3)	**Mean**	8.80 ^a^	5.01 ^a^	5.50 ^a^
	**SD**	8.10	4.33	4.86
*Festuca arundinacea* ’Kora’ (n = 3)	**Mean**	8.79 ^a^	4.87 ^a^	4.99 ^a^
	**SD**	8.07	4.46	2.36
*Lolium perenne* ’Promed’ (n = 3)	**Mean**	10.14 ^d^	3.88 ^a^	5.82 ^a^
	**SD**	8.64	3.53	5.54
Mixture I (n = 3)	**Mean**	8.86 ^ab^	5.95 ^a^	6.13 ^a^
	**SD**	7.66	5.34	5.10
Mixture II (n = 3)	**Mean**	9.18 ^b^	6.97 ^b^	7.04 ^b^
	**SD**	8.45	6.51	6.51
Mixture III (n = 3)	**Mean**	9.47 ^c^	6.78 ^ab^	6.34 ^a^
	**SD**	8.59	6.58	4.96

Results are expressed as a mean and standard deviation (SD). Mean values are statistically significant at p < 0.05, when values in columns are marked with a different letter in the upper index.

Highest number of yeasts was found in Mixture II (9.26 CFU g ^-1^), lowest in ryegrass (7 500 CFU g ^-1^), there were no significant differences between Mixtures I, Mixture III and the monocultures. Moreover, significantly higher amount of OF was found in Mixture II (10 915 151 CFU g ^-1^) in comparison to the other variants. Lowest OF contamination was found in ’Aprilia’ (5.80x10^7^ CFU g ^-1^).

In mycotoxin testing, average DON concentration was below the limit of detection in our monoculture samples, in mixtures it was 1.76 μg kg^-1^. ZEN levels of monocultures were significantly lower in comparison to species-rich mixtures, 0.42 μg kg^-1^ and 0.87 μg kg^-1^ respectively. However, AFB1 levels were significantly higher in monocultures (0.05 μg kg^-1^) in comparison to mixtures (0.03 μg kg^-1^).

No significant differences were found between ZEN and AFB1 levels of each individual variant (Table 5). Highest average ZEN levels were found in Mixture II (0.91 μg g ^-1^), lowest in ’Kora’ (0.30 μg g ^-1^). AFB1 levels were significantly lower in Mixture II and III in comparison to Mixture I, ryegrass and fescues. The highest AFB1 contamination was found in ’Aprilia’ (18.33 μg g ^-1^), the lowest in Mixture III (4 μg g ^-1^).

**Table 5:** Average mycotoxin contamination depending on botanical species.

		Deoxynivalenol (μg kg^-1^)	Aflatoxin B1 (μg kg^-1^)	Zearalenone (μg kg^-1^)
*Festuca arundinacea* ’Prostela’ (n = 3)	**Mean**	<LOQ	0.0502 ^a^	0.4862 ^a^
	**SD**	<LOQ	0.0016	0.4406
*Festuca arundinacea* ’Aprilia’ (n = 3)	**Mean**	<LOQ	0.0520 ^a^	0.3810 ^a^
	**SD**	<LOQ	0.0024	0.0706
*Festuca arundinacea* ’Philia’ (n = 3)	**Mean**	<LOQ	0.0500 ^a^	0.3620 ^a^
	**SD**	<LOQ	0.0070	0.0613
*Festuca arundinacea* ’Kora’ (n = 3)	**Mean**	<LOQ	0.0521 ^a^	0.2955 ^a^
	**SD**	<LOQ	0.0038	0.0137
*Lolium perenne* ’Promed’ (n = 3)	**Mean**	<LOQ	0.0420 ^a^	0.5896 ^a^
	**SD**	<LOQ	0.0012	0.0271
**Mixture I (n = 3)**	**Mean**	1.3307	0.0535 ^a^	0.8930 ^a^
	**SD**	0.2061	0.0349	0.6261
**Mixture II (n = 3)**	**Mean**	2.7228	0.0212 ^a^	0.9140 ^a^
	**SD**	1.9976	0.0165	0.1759
**Mixture III (n = 3)**	**Mean**	1.2348	0.0229 ^a^	0.8082 ^a^
	**SD**	0.8555	0.0099	0.1832

Results are expressed as a mean and standard deviation (SD). Mycotoxin concentration marked as <LOQ were under the limit of quantification (DON < 0.03 μg kg^-1^). Mean values are statistically significant at p < 0.05, when values in columns are marked with a different letter in the upper index.

## Discussion

Effects of acute or chronic mycotoxin toxicity can cause significant economic losses by decreasing feed efficiency, immune competence, and overall performance [13]. World annual crop losses of 1 billion metric tons have been estimated by Food and Agriculture Organization of the United (FAO) [14]. Hassan and Zhou [15] estimate that annual losses due to fungal infections and crop contamination can be as high as US$ 5 billion in the North America. Furthermore, these authors state that costs connected to mycotoxin occurrence in both food and feed will continue to rise. Marasas et al. [13] states, that estimated mean annual cost related to food and feed contamination by DON, aflatoxin and fumonisin damage in the USA is US$ 946 million, with over US$ 100 million for aflatoxins alone [16].

Infection of feed crops with mycotoxigenic fungal pathogens is a worldwide problem. The strict limits of aflatoxin concentration are defined for food in Commission recommendation 2005/925/EC to protect human health. In feed, the Directive 2002/32/EC on undesirable substances in animal feed regulates maximum limits of aflatoxins in feed [17]. However, there are no actual limits set by the European Commission (EC) for fresh forage contamination in general. In case of AFB1, it may be due to low incidence of aflatoxins in the EU, however for other mycotoxins (ochratoxin A, DON, fumonisins, T-2 toxin, HT-2 toxin, ZEN and patulin) there are no limits either. On the other hand, Council Directive 95/53*/*EC recommends monitoring their occurrence in feed [18]. In the newer EC recommendation from 2006, guidance for maximum concentration of several mycotoxins has been established; however, hay or fresh forage limits were not included [19]. In comparison to these EC recommendations, limits for DON (0.9–12 μg kg^-1^) or ZEN (0.25–3 μg kg^-1^) were not exceeded in this experiment.

Interaction between several factors, such as year and plant density, may also affect mycotoxin content [20]. These interactions may explain differences between mixtures and monocultures. This is supported by the work of Zachariasova et al. [21], where DON, ZEN and aflatoxins were not detected in clover-grass forage samples. However, DON and ZEN did occur in hay. No differences in concentration of DON, ZEN and aflatoxins were found between perennial ryegrass monoculture and fescue-based mixture by Skladanka et al. [22]. Our results showed no significant differences between monoculture species and therefore are in agreement with their study. However, Baholet et al. [23] found significantly higher DON and ZEN contamination in samples of tall fescue than perennial ryegrass. These conflicting results may be caused by interannual variability that occurs in phyllosphere as well as mycotoxin contamination [24, 25].

Phenotype as well as genotype affect the phyllosphere composition [26, 27]. Burkin et al. [4] stated that the amount of mycotoxin in a monoculture depends on the monoculture species. Pieczul et al. [28] studied mycoflora on *Arhenantherum aristatum*; this species, related to *A*. *odoratum* present in our experimental mixture, is infected by many fungi pathogen species, of which *Fusarium* spp. Is the most important. Presence of this species could therefore increase the risk of high OF counts and subsequent mycotoxin contamination in Mixture 3. Out of 26 identified species found on Polish meadow was the highest level of fungal contamination found in tall fescue and perennial ryegrass, which suggests potential feed safety risk of these meadow species [3]. However, results of our experiment did not find microbial differences between the grass species’ amounts of fungi and yeasts. This is in accordance with Baholet et al. [23], where no significant differences were observed between ryegrass, fescue and timothy. On the contrary, TMC in our experiment was significantly higher in ryegrass in comparison to fescues, which can suggest effects of other factors on the epiphytic microbial communities. Results of Seguin et al. [29] showed, that both fungal species and fungal counts in the sample were highly dependent on the botanical species sampled. Higher fungal counts were observed more often in monoculture hay in comparison with multi-species hay, however, these results depend on the type of multi-species hay observed. This was supported especially in case of significantly lower fungal contamination was found in perennial ryegrass hay in comparison with *Alopecurus geniculatus* or Crau and Swiss hay. Bacterial communities have also been proven to show species-specific differences in amounts [30]. Epiphytic microbiota is considered an important quality factor in subsequent processing of biomass into conserved feed [30, 31]. Authors therefore imply that besides floristic composition of a sample and geographical location significantly affect hygienic quality of hay.

Biomass gathered from meadows can be colonized by multiple species of fungal mycotoxin producers, including *Humicola* spp., *Alternaria* spp., *Cladosporium* spp., *Torula* spp., *Fusarium* spp. and *Mucor* spp. [3]. Birdsfoot trefoil (*Lotus corniculatus* L.) is a host plant of the genera *Alternaria*, *Phytophthora*, *Sclerotinia*, *Bipolaris*, *Rhizoctonia* and *Fusarium*, while *Fusarium oxysporum* being one of the most important pathogens of legumes [32, 33]. Accensi et al. [34] compared three types of feed samples and found that higher occurrence of *Aspergillus* spp., which also include the main producer of aflatoxins *Aspergillus flavus*, was detected in the legumes (94.4%) in comparison to mixed feed samples (89.8%) and cereals (57.3%) [35]. Their findings suggest that higher AFB1 concentration would potentially occur in species-rich mixtures containing legumes than in grass monocultures. However, this was not in accordance with the results obtained in this experiment. AFB1 concentration were significantly higher in monocultures than in mixtures containing legumes. Furthermore, mixtures that contained lower percentage of legumes (Mixture 2 and Mixture 3) did not differ from high legume Mixture 1 in the concentration of AFB1.

Crops have a different level of susceptibility to infection by mycotoxigenic fungi and consequently to mycotoxin contamination [36]. Presence of mycotoxins in key fodder crops such as maize as well as in small grain cereals and legumes e.g. pea and soy is well known [21, 37, 38]. Infection by toxicogenic fungi depends on many abiotic factors, such as weather conditions, nutrient deficiency or excess, and biotic factors, such as insect damage. All of these factors predispose plant biomass for contamination in field [39]. Late forage harvest may lead to higher contamination by microscopical fungi and thus to higher levels of mycotoxins [22, 39]. This is in accordance with the study of Schenck et al. [40], where OF counts were higher in samples with higher dry matter content. It is possible to assume that species-rich mixtures will be usually harvested later than grass monocultures, so that they can fulfill their ecosystem services. This hypothesis may partially explain the higher concentration of DON and ZEN in mixtures in comparison to monocultures. However, it does not support this hypothesis in terms of AFB1 contamination.

Plant genotype is a factor affecting the phyllospheric microbial communities differently, depending on the observed microbial group [41]. When evaluated, forage crops grown in agroforestry systems showed similar levels of fungal colonization and subsequent mycotoxin contamination as traditional monocultures. This may indicate the importance of species-rich stands in terms of ecosystem services, without significant negative effects on the fungal contamination of the final product [9]. In our study, there were significant differences among species-rich mixtures in terms of microbial counts. Even between Mixture II and III, where the species composition was the same, some differences were found. The abundance of species in the mixtures differed, however. Due to this we hypothesize, that the reason is that one or multiple components, that are highly colonized by yeasts and fungi and, might have caused high CFU counts in the experiment.

Plant defense mechanisms may be affected by amounts of specific lipids, such as sphingolipids in cell membrane [42] or cell wall [43]. Zachariasova et al. [21], however, state that maize leaves and corncobs contain higher amounts of protein and polysaccharides than grass, therefore they provide suitable conditions for surviving and spreading of fungi and other microorganisms. Moreover, in the study of Venslovas et al. [38], negative correlation between crude protein and crude fiber levels as well as ZEN concentration in silage samples was found. Higher percentage of protein-rich components occur in legumes species. This may be one of the factors responsible for higher OF counts as well as DON and ZEN contamination in mixtures, where legume component was present, in comparison to monocultures. This theory, however, does not explain why Mixture 2 has higher OF counts in comparison to Mixture 1, where the legume component has higher percentage overall. This points to other factors affecting mycotoxin production. Similarly to our experiment, Schenck et al. [40] did not find any correlation between organic nutrients and OF counts or mycotoxin concentrations. On the other hand, Venslovas et al. [38] found that DON concentration was negatively correlated with dry matter content of grass silage.

## Conclusion

Due to the current research and legislation focus on food and feed safety, the relationship between agricultural commodities, microbial populations and subsequent mycotoxin contamination influences the evaluation of potential health complications in animals. Contaminated animal products can serve as a mycotoxin source in foodstuffs and cause long term health problems. Therefore, our research helps to start a crucial conversation between consumers, farmers and ecologists and establish good practices in diversity conservation as well as forage production. However, there are multiple factors involved in microbial colonization of phyllosphere and we cannot unambiguously say that a monocultures or species-rich stands are safer in terms of mycotoxin producers or mycotoxin contamination.

## Supporting information

S1 Dataset (XLSX)

## References

[1] Sala OE, Vivanco L, Flombaum P. Grassland Communities and Ecosystems☆. Reference Module in Life Sciences. Elsevier; 2017. doi: 10.1016/B978-0-12-809633-8.02201-9

[2] Fleury P, Seres C, Dobremez L, Nettier B, Pauthenet Y. “Flowering Meadows”, a result-oriented agri-environmental measure: Technical and value changes in favour of biodiversity. Land Use Policy. 2015;46: 103–114. doi: 10.1016/j.landusepol.2015.02.007

[3] Twarużek M, Dembek R, Pańka D, Soszczyńska E, Zastempowska E, Grajewski J. Evaluation of Cytotoxicity and Mould Contamination of Selected Plants from Meadows Covered by the Agri-Environmental Program. Toxins. 2019;11: 228. doi: 10.3390/toxins11040228 30999701 PMC6520750

[4] Burkin AA, Kononenko GP. Mycotoxin Contamination of Meadow Grasses in European Russia. Selskokhozyaistvennaya Biol. 2015;50: 503–512. doi: 10.15389/agrobiology.2015.4.503eng

[5] The European Medicines Agency. Committee for Medicinal Products Veterinary Use (CVMP) Meeting of 14–16 March 2017. Sep 17, 2018. Available: https://www.ema.europa.eu/en/news/committee-medicinal-products-veterinary-use-cvmp-meeting-14-16-march-2017

[6] Bragulat MR, Martínez E, Castellá G, Cabañes FJ. Ochratoxin A and citrinin producing species of the genus Penicillium from feedstuffs. Int J Food Microbiol. 2008;126: 43–48. doi: 10.1016/j.ijfoodmicro.2008.04.034 18571755

[7] Medina Á, González-Jartín JM, Sainz MJ. Impact of global warming on mycotoxins. Food Toxicol • Food Saf. 2017;18: 76–81. doi: 10.1016/j.cofs.2017.11.009

[8] Kononenko GP, Gavrilova OP, Gagkaeva TY. Fungal species and multiple mycotoxin contamination of cultivated grasses and legumes crops. Agric Food Sci. 2015;24: 323–330.

[9] Beule L, Lehtsaar E, Rathgeb A, Karlovsky P. Crop Diseases and Mycotoxin Accumulation in Temperate Agroforestry Systems. Sustainability. 2019;11: 2925. doi: 10.3390/su11102925

[10] Wu F. Measuring the economic impacts of Fusarium toxins in animal feeds. Anim Feed Sci Technol. 2007;137: 363–374. doi: 10.1016/j.anifeedsci.2007.06.010

[11] Rekha C, Shridhar NB, Jagadeesh SS, Narayanaswamy HD. Isolation and identification of fungal isolates from contaminated meadow grass fodder. J Livestock Sci. 2015;6: 104–108.

[12] Gallo A, Giuberti G, Frisvad JC, Bertuzzi T, Nielsen KF. Review on Mycotoxin Issues in Ruminants: Occurrence in Forages, Effects of Mycotoxin Ingestion on Health Status and Animal Performance and Practical Strategies to Counteract Their Negative Effects. Toxins. 2015;7: 3057–3111. doi: 10.3390/toxins7083057 26274974 PMC4549740

[13] Marasas WFO, Gelderblom W, Shephard G, Vismer H. Mycotoxins: A global problem. Mycotoxins Detect Methods Manag Public Health Agric Trade. 2008; 29–39. doi: 10.1079/9781845930820.0029

[14] Schmale DG, Munkvold GP. Mycotoxins in Crops ‐ A Threat to Human and Domestic Animal Health. Plant Health Instr. 2014 [cited 9 Feb 2022]. Available: https://www.apsnet.org/edcenter/disimpactmngmnt/topc/Mycotoxins/Pages/default.aspx

[15] Hassan YI, Zhou T. Promising Detoxification Strategies to Mitigate Mycotoxins in Food and Feed. Toxins. 2018;10: 116. doi: 10.3390/toxins10030116 29522477 PMC5869404

[16] Coulibaly O, Hell K, Ranajit Bandyopadhyay RB, Hounkponou S, Leslie JF. Economic impact of aflatoxin contamination in sub-Saharan Africa. Mycotoxins Detect Methods Manag Public Health Agric Trade. 2008; 67–76. doi: 10.1079/9781845930820.0067

[17] European Commission. Directive 2002/32/EC of the European Parliament and of the Council of 7 May 2002 on undesirable substances in animal feed ‐ Council statement. OJ L May 7, 2002. Available: http://data.europa.eu/eli/dir/2002/32/oj/eng

[18] European Commission M. Commision Recommendation on the coordinated inspection programme in the field of animal nutrition for the year 2006 in accordance with Council Directive 95/53/EC. 2005 [cited 15 Jul 2022]. Available: https://eur-lex.europa.eu/legal-content/EN/TXT/HTML/?uri=CELEX:32005H0925&from=CS

[19] European Commission. Commission Recommendation of 17 August 2006 on the presence of deoxynivalenol, zearalenone, ochratoxin A, T-2 and HT-2 and fumonisins in products intended for animal feeding. OJ L. 2006 Aug. Report No.: 32006H0576. Available: http://data.europa.eu/eli/reco/2006/576/oj/eng

[20] Krnjaja V, Mandić V, Stanković S, Obradović A, Vasić T, Lukić M, et al. Influence of plant density on toxigenic fungal and mycotoxin contamination of maize grains. Crop Prot. 2019;116: 126–131. doi: 10.1016/j.cropro.2018.10.021

[21] Zachariasova M, Dzuman Z, Veprikova Z, Hajkova K, Jiru M, Vaclavikova M, et al. Occurrence of multiple mycotoxins in European feedingstuffs, assessment of dietary intake by farm animals. Anim Feed Sci Technol. 2014;193: 124–140. doi: 10.1016/j.anifeedsci.2014.02.007

[22] Skladanka J, Adam V, Dolezal P, Nedelnik J, Kizek R, Linduskova H, et al. How Do Grass Species, Season and Ensiling Influence Mycotoxin Content in Forage? Int J Environ Res Public Health. 2013;10: 6084–6095. doi: 10.3390/ijerph10116084 24225645 PMC3863888

[23] Baholet D, Kolackova I, Kalhotka L, Skladanka J, Haninec P. Effect of Species, Fertilization and Harvest Date on Microbial Composition and Mycotoxin Content in Forage. Agriculture. 2019;9: 102. doi: 10.3390/agriculture9050102

[24] Bao L, Gu L, Sun B, Cai W, Zhang S, Zhuang G, et al. Seasonal variation of epiphytic bacteria in the phyllosphere of Gingko biloba, Pinus bungeana and Sabina chinensis. FEMS Microbiol Ecol. 2020;96: fiaa017. doi: 10.1093/femsec/fiaa017 32005997

[25] Benešová K, Boško R, Běláková S, Pluháčková H, Křápek M, Pernica M, et al. Natural contamination of Czech malting barley with mycotoxins in connection with climate variability. Food Control. 2022;140: 109139. doi: 10.1016/j.foodcont.2022.109139

[26] Li Y, Wu X, Chen T, Wang W, Liu G, Zhang W, et al. Plant Phenotypic Traits Eventually Shape Its Microbiota: A Common Garden Test. Front Microbiol. 2018;9. Available: https://www.frontiersin.org/article/10.3389/fmicb.2018.02479 30459725 10.3389/fmicb.2018.02479 PMC6232875

[27] Sun A, Jiao X-Y, Chen Q, Wu A-L, Zheng Y, Lin Y-X, et al. Microbial communities in crop phyllosphere and root endosphere are more resistant than soil microbiota to fertilization. Soil Biol Biochem. 2021;153: 108113. doi: 10.1016/j.soilbio.2020.108113

[28] Pieczul K, Swierczynska I, Byczkowska K, Drapikowska M. Preliminary research on pathogenic fungi colonizing Anthoxanthum aristatum Boiss. Pak J Bot. 2022;54. doi: 10.30848/PJB2022-4(12)

[29] Seguin V, Lemauviel-Lavenant S, Garon D, Bouchart V, Gallard Y, Blanchet B, et al. An evaluation of the hygienic quality in single-species hays and commercial forages used in equine nutrition. Grass Forage Sci. 2010;65: 304–317. doi: 10.1111/j.1365-2494.2010.00751.x

[30] Wang S, Shao T, Li J, Zhao J, Dong Z. Fermentation Profiles, Bacterial Community Compositions, and Their Predicted Functional Characteristics of Grass Silage in Response to Epiphytic Microbiota on Legume Forages. Front Microbiol. 2022;13. Available: doi: 10.3389/fmicb.2022.830888 35211107 PMC8861195

[31] Wang S, Zhao J, Dong Z, Li J, Kaka NA, Shao T. Sequencing and microbiota transplantation to determine the role of microbiota on the fermentation type of oat silage. Bioresour Technol. 2020;309: 123371. doi: 10.1016/j.biortech.2020.123371 32305853

[32] Jha UC, Bohra A, Pandey S, Parida SK. Breeding, Genetics, and Genomics Approaches for Improving Fusarium Wilt Resistance in Major Grain Legumes. Front Genet. 2020;11. Available: https://www.frontiersin.org/articles/10.3389/fgene.2020.01001 33193586 10.3389/fgene.2020.01001 PMC7644945

[33] Vasić T, Krnjaja V, Marković J, Andjelković S, Petrović M, Leposavić A, et al. Fungal pathogens of birdsfoot trefoil (Lotus corniculatus L.) in Serbia. Proc X Int Sci Agric Symp “Agrosim 2019.” 2019; 1025–1029.

[34] Accensi F, Abarca ML, Cabañes FJ. Occurrence of Aspergillus species in mixed feeds and component raw materials and their ability to produce ochratoxin A. Food Microbiol. 2004;21: 623–627. doi: 10.1016/j.fm.2003.12.003

[35] ANSES. Aspergillus flavus and other aflatoxin-producing moulds. ANSES: French Agency for Food, Environmental and Occupational Health & Safety; 2013. Available: https://www.anses.fr/en/system/files/MIC2012sa0053FiEN.pdf

[36] Rose LJ, Okoth S, Flett BC, Rensburg BJ van, Viljoen A. Preharvest Management Strategies and Their Impact on Mycotoxigenic Fungi and Associated Mycotoxins. Mycotoxins ‐ Impact and Management Strategies. IntechOpen; 2018. doi: 10.5772/intechopen.76808

[37] Garcia D, Barros G, Chulze S, Ramos AJ, Sanchis V, Marín S. Impact of cycling temperatures on Fusarium verticillioides and Fusarium graminearum growth and mycotoxins production in soybean. J Sci Food Agric. 2012;92: 2952–2959. doi: 10.1002/jsfa.5707 22555960

[38] Venslovas E, Merkeviciute-Venslove L, Mankeviciene A, Kochiieru Y, Slepetiene A, Ceseviciene J. The prevalence of mycotoxins and their relation to nutrient composition of maize and grass silage. Zemdirb-Agric. 2021;108: 147–152. doi: 10.13080/z-a.2021.108.019

[39] Prandini A, Sigolo S, Filippi L, Battilani P, Piva G. Review of predictive models for Fusarium head blight and related mycotoxin contamination in wheat. Food Chem Toxicol. 2009;47: 927–931. doi: 10.1016/j.fct.2008.06.010 18634842

[40] Schenck J, Müller C, Djurle A, Jensen DF, O’Brien M, Johansen A, et al. Occurrence of filamentous fungi and mycotoxins in wrapped forages in Sweden and Norway and their relation to chemical composition and management. Grass Forage Sci. 2019;74: 613–625. doi: 10.1111/gfs.12453

[41] Whipps JM, Hand P, Pink D, Bending GD. Phyllosphere microbiology with special reference to diversity and plant genotype. J Appl Microbiol. 2008;105: 1744–1755. doi: 10.1111/j.1365-2672.2008.03906.x 19120625

[42] Iqbal N, Czékus Z, Poór P, Ördög A. Plant defence mechanisms against mycotoxin Fumonisin B1. Chem Biol Interact. 2021;343: 109494. doi: 10.1016/j.cbi.2021.109494 33915161

[43] Giancaspro A, Lionetti V, Giove SL, Zito D, Fabri E, Reem N, et al. Cell wall features transferred from common into durum wheat to improve Fusarium Head Blight resistance. Plant Sci. 2018;274: 121–128. doi: 10.1016/j.plantsci.2018.05.016 30080595

